# Recognizing Disguised Faces: Human and Machine Evaluation

**DOI:** 10.1371/journal.pone.0099212

**Published:** 2014-07-16

**Authors:** Tejas Indulal Dhamecha, Richa Singh, Mayank Vatsa, Ajay Kumar

**Affiliations:** 1 IIIT-Delhi, New Delhi, India; 2 Department of Computing, The Hong Kong Polytechnic University, Hung Hom, Hong Kong SAR, China; Plymouth University, United Kingdom

## Abstract

Face verification, though an easy task for humans, is a long-standing open research area. This is largely due to the challenging covariates, such as disguise and aging, which make it very hard to accurately verify the identity of a person. This paper investigates human and machine performance for recognizing/verifying disguised faces. Performance is also evaluated under familiarity and match/mismatch with the ethnicity of observers. The findings of this study are used to develop an automated algorithm to verify the faces presented under disguise variations. We use automatically localized feature descriptors which can identify disguised face patches and account for this information to achieve improved matching accuracy. The performance of the proposed algorithm is evaluated on the IIIT-Delhi Disguise database that contains images pertaining to 75 subjects with different kinds of disguise variations. The experiments suggest that the proposed algorithm can outperform a popular commercial system and evaluates them against humans in matching disguised face images.

## Introduction

The pursuit to find the most accurate face representation and perform recognition has passed through shifts in the research *paradigms* used [1] as well as shifts in the *challenges* addressed. (In this paper, the term recognition and verification are interchangeably used.) Some major approaches proposed for face recognition, in chronological order (but not limited to), are Principal Component Analysis (PCA) [2], Fisher's Linear Discriminant Analysis (LDA) [3], Independent Component Analysis (ICA) [4], Elastic Bunch Graph Matching (EBGM) [5], Local Binary Patterns (LBP) [6], Scale Invariant Feature Transform (SIFT) [7], and Sparse Representation Classifier (SRC) [8]. Earlier research has primarily focused on the challenges or covariates of pose, illumination and expression whereas recently, face alterations due to plastic surgery [9], sketch-to-photo matching [10], [11], multi-spectrum matching [12]–[14], aging [15]–[17], and disguise [18]–[20] are also being explored. Given the current state of automated face recognition algorithms [21], it is likely that in the near future, automated face recognition will be used for controlled applications such as access control and attendance systems, and as one of the modalities in adverse environment applications such as law enforcement. Currently, state-of-the-art systems including commercial systems have shown excellent performance with limited challenges of pose, illumination, and expression [21]. However, in presence of emerging covariates, the performance of state-of-the-art systems have not been studied extensively. This research focuses on understanding the face recognition performance of humans and then incorporating these findings to design an algorithm for recognizing disguised faces.

**Figure 1:**
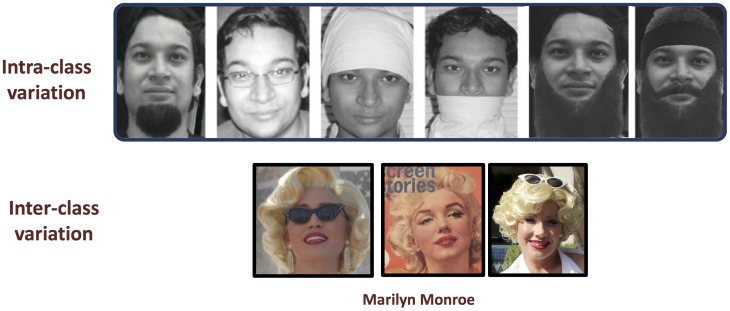
Illustrating the effect of disguise accessories on inter-class and intra-class variations. Top row images pertain to one of the authors (MV) and bottom row images are taken from Internet under Creative Commons Attribution (CC BY) license (Original images, source weblinks and attributions are given in Supporting Information S1).

*Disguise* is an interesting and a challenging covariate of face recognition. It involves both intentional and unintentional changes on a face through which one can either obfuscate his/her identity and/or impersonate someone else's identity. In either case, facial disguise falls under the broader category of *biometric obfuscation* [22]. Figure 1 shows an example of *face obfuscation* where the appearance of a subject can vary by using different disguise accessories. (Note that the images in Figure 1 may be affected by covariates other than disguise, e.g. aging; however, in this work we are concentrating on disguise only). As shown in Figure 1, disguise increases the *intra-class* variation (when it is used to hide one's identity) and reduces the *inter-class* variation (when it is used to impersonate someone else). Even though the problem of face recognition under disguise is prevalent in real world applications, it has not been studied extensively. To make automatic face recognition more usable and secure, it is necessary to address the problem of (at least unintentional) disguise.

In recent years, recognition of disguised faces by humans has been an interesting area of research for cognitive scientists. Righi et al. [23] studied the effect of adding or removing the disguise accessories such as wigs and eyeglasses. They also evaluated the *switch*/*no switch* scenario where the accessories present during training phase were removed (switch) or kept unaltered (no switch). The study revealed that increasing the alterations to facial attributes of the probe image decreased the recognition performance. Further, the change in the rather stable facial features such as eyes had comparatively higher impact in decreasing the performance. A more detailed analysis regarding the effect of disguise on eye region was presented in [24], [25]. Sinha et al. [24] studied the importance of eye brows stating “Of the different facial features, eyebrows are among the most important for recognition”. Douma et al. [25] found that removing glasses during testing had more damaging effect than adding; this is also called as the Clark-Kent effect [26]. The authors did not find any significant effect of familiarity on recognition. However, familiarizing the participant nine times did show significant performance difference than familiarizing three times. At a level of abstraction, Sinha et al. [24] and Douma et al. [25] provided insights about the effect of disguise on stable features. Complimentarily, the effect of hair – rather unstable features – was studied by Toseeb et al. [27]. The authors observed no significant performance difference when the participants were shown faces with and without hair. The phenomenon was attributed to the *internal face features*, which remained constant in both the scenarios. Similarly, the effect of internal features was also studied in [28], [29]. Overall, it appears that the effect of disguise on stable facial parts has more impact than on the unstable facial parts. However, to the best of our knowledge, a comprehensive research on the effect of disguising individual facial parts and their combinations is not performed.

Since disguise can be viewed as alteration to visual face information, the research related to recognition of altered/degraded facial images can potentially provide some insights. In presence of image degradation by blurring, Sinha et al. [24] have shown that familiarity of the stimuli subjects is advantageous for face recognition. Complimentarily, Hancock et al. [30] reported that unfamiliar faces are difficult to recognize in a low-quality surveillance video. Combining their results [24], [30] point to a possibility that the representation of familiar faces might be more robust to certain image degradations than that of unfamiliar faces. Therefore, understanding the effect of familiarity on disguised face recognition can potentially provide insights into the robust facial representation and recognition by humans. It has been also observed in literature that face recognition by humans is subjective to familiarity [31] and race [32].

A brief overview of literature related to automated face recognition under disguise variations is presented in Table 1. Note that most of the research has been performed using the AR [33] and Yale [34] face databases which contain very limited disguise (sunglasses and scarves only). However, to be confident about the performance of automated approaches, it is required that evaluation is performed on a dataset with more exhaustive disguise variations. Regarding the effect of ethnicity, Phillips et al. [35] evaluated the performance of algorithms on east Asian and Caucasian faces. The study showed that the fusion of the algorithms developed in east Asia performed better on east Asian faces than on Caucasian faces. Similarly, fusion of the algorithm developed in West countries performed better on Caucasian faces than east Asian faces.

**Table 1:** Literature review.

Authors	Algorithm	Disguise detection	Disguise/occlusion detected as	Face recognition	Spectrum	Database
Ramanathan et al. [18]	PCA	Yes	Left/right half face	Yes	Visible	National Geographic, AR
Singh et al. [19]	2D-log polar Gabor	No	-	Yes	Visible	AR, Private 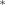 , Synthetic Disguise 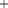
Martinez [53]	Probabilistic matching	No	-	Yes	Visible	AR
Wright et al. [8]	SRC	No	-	Yes	Visible	AR, Yale B [34]
Kim et al. [54]	ICA	No	-	Yes	Visible	AR, FERET
Yang and Zhang [55]	Gabor SRC	No	-	Yes	Visible	AR, Yale B
Pavlidis and Symosek [44]	-	Yes	Not explicitly	No	Near-IR	-
Yoon and Kee [56]	PCA + SVM	Yes	Upper/lower half	No	Visible	AR, Private 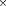
Kim et al. [57]	PCA + SVM	Yes	Upper/lower half	No	Visible	AR, Private 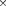
Choi and Kim [58]	AdaBoost + MCT-based features	Yes	Left-right eye, mouth	No	Visible	AR
Min et al. [49]	Gabor + PCA + SVM, LBP	Yes (Gabor + PCA + SVM)	Upper/lower half	Yes (LBP)	Visible	AR
Dhamecha et al. [20]	ITE, LBP	Yes (ITE)	Individual patches	Yes (LBP)	Visible and Thermal	I 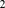 BVSD

Existing algorithms for addressing disguise variations. AR database [33] contains 3200+ images pertaining to 126 subjects with two kinds of disguises (sunglasses and scarves). The National Geographic (NG) dataset contains 46 images of 1 individual, with various accessories such as hat, glasses, sunglasses, and facial hair. *Private dataset of 150 images pertaining to 15 individuals which contains similar real and synthetic disguise variations as in NG dataset. 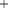
Synthetic disguise dataset of 4000 images pertaining to 100 individuals. 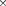
Private datasets are collected by researches in real world scenarios from ATM (automatic teller machine) kiosk.

In the last decade, some studies compared the performance of automated face recognition algorithms and humans. O′Toole et al. [36] compared human performances with academic and commercial systems. They observed that on the *easy* pairs, all the automated algorithms, except one, exhibited better performance than humans; while for the *difficult* pairs, some algorithms outperformed humans. This study focused on understanding the effects of the illumination variation and, interestingly, the image pairs that were *difficult* for PCA based algorithms were also found to be difficult for humans. Moreover, the evidences of algorithms surpassing humans for face verification task were also observed. Similar comparison was presented in [37] for face recognition under uncontrolled illumination, indoor and outdoor settings, and day-to-day appearance variation. In [37], algorithms were shown to have superior performance than humans for *good* and *moderate* image pairs, whereas humans and algorithms were comparable for the *poor* accuracy group. These good, moderate, and poor accuracy groups were created based on scores given by algorithms. Though not for face recognition, but for face detection, Marius't [38] reported the *similar-error* phenomena by humans and automated algorithm (AdaBoost cascade classifier [39]). Further, O′Toole et al. [40] fused the humans and algorithms for face verification task using partial least square regression. The fusion resulted in significant performance improvement. To the best of our knowledge, neither 1) a study focusing on covariate of disguise has been carried out, nor 2) any attempt to enhance machine performance by encoding human strategy for recognizing disguised faces has been made.

In this research we evaluate the effect of familiarity and ethnicity on disguised face recognition, and attempt to encode learnings from human evaluations into an automated algorithm. Since humans are considerably efficient at face recognition [36], comparison of humans and automated algorithms is also performed. The main contributions from this research can be summarized as follows:

- evaluating human face recognition performance under face disguise along with familiarity and ethnicity/race effect;
- determining the effect of individual facial parts on the overall human face recognition performance;
- proposing an automated face recognition algorithm based on the learnings from human evaluation and comparing the performance with SRC [8] and a commercial off-the-shelf (COTS) system; and
- comparison of human performance with automated algorithms (including the proposed algorithm) for addressing disguise variations.

## Materials and Methods

### Ethics

To undertake this research the first step was to create a database. At the time of database creation all the 75 subjects in the database were of age 18+ years. The subjects were provided with accessories, and were asked to use the accessories at their will in order to get disguised. All the subjects provided written informed consent for using their face images for research purpose. The consent, for sharing their face images with research community and publish their face images in research papers, was also taken from the subjects. Images pertaining to only those subjects who gave their consent for sharing their face images, will be made available to the research community.

**Figure 2:**
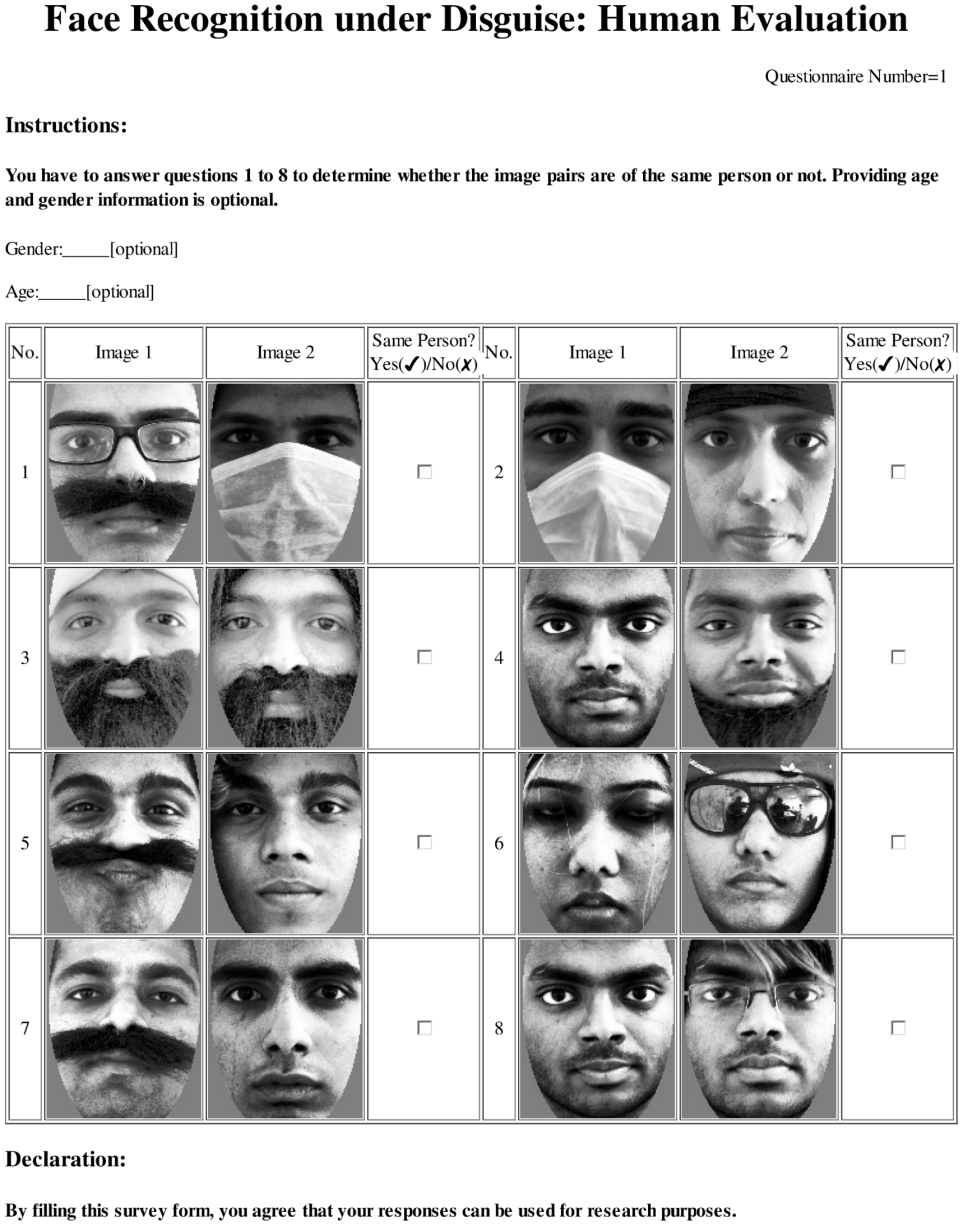
Sample questionnaire.

In order to analyze human capability of recognizing disguised faces, we collected the responses from various participants. All the responses collected from survey participants are anonymous and are used only for research purposes. Their willingness to participate in the survey was also asked. A sample survey collection form is shown in Figure 2. The database collection and survey response collection procedures for this study were approved by the IIIT-Delhi Ethics Board.

### Disguise Face Database

The databases generally used for disguise related research (AR [33] and Yale [34] face databases) contain very limited disguise variations, such as scarves and/or sun-glasses. Therefore, to evaluate the effectiveness of automated algorithms and to evaluate human performance, we have collected the IIIT-Delhi Disguise Version 1 face database (ID V1) of disguised/obfuscated face images. The ID V1 database contains 681 visible spectrum images of 75 participants (all above the age of 18 years) with disguise variations. The number of images per person varies from 6 to 10. For every subject, there is at least one frontal neutral. Here, face image without any disguise is referred as *neutral* face image. face image and at least five frontal disguised face images. All the face images are captured under (almost) constant illumination with neutral expression and frontal pose. The disguise variations included in the database are categorized into the following categories.

- **Without disguise**: neutral image,
- **Variations in hair style**: different styles and colors of wigs,
- **Variations due to beard and mustache**: different styles of beards and mustaches,
- **Variations due to glasses**: sunglasses and spectacles,
- **Variations due to cap and hat**: different kinds of caps, turbans, veil (also known as hijab which covers hair), and bandanas,
- **Variation due to mask**: disposable doctors mask, and
- **Multiple variations**: a combination of multiple disguise accessories.

**Figure 3:**
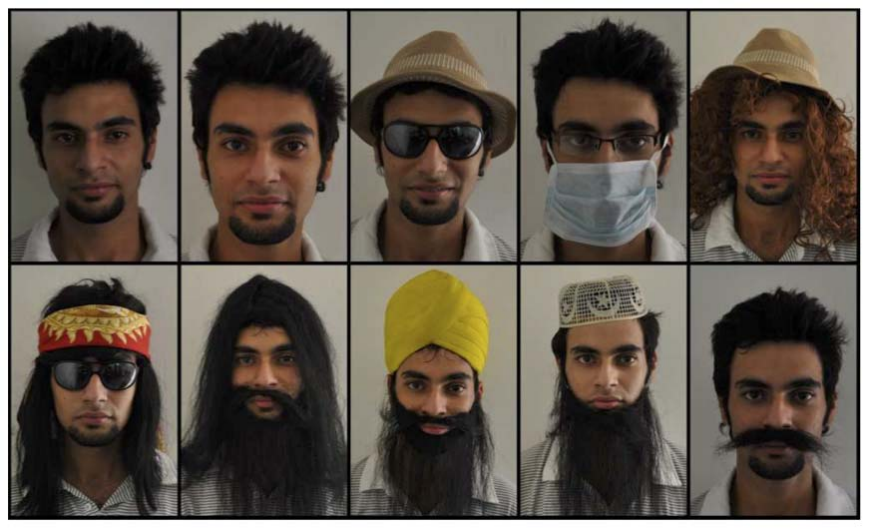
Sample images from the ID V1 database.

Figure 3 shows sample images from the database. The disguises are chosen in such a way that they result in more realistic appearances and (almost) every part of the face is hidden at least once. The subjects are asked to disguise themselves using the given accessories. This allows different subjects to have different types of disguises thus providing more variations across individuals in the database. The database is publicly available for research purpose [41]. The images from the dataset are preprocessed in the same way as in [20] i.e. preprocessing is done using the CSU Face Identification Evaluation System [42] to obtain normalized images.

### Participants for Human Evaluation

Since this study examines the effect of ethnicity and familiarity factors on face recognition with disguise variations, the participants were divided into the following four sets.

- **Set 1**: familiar to the subjects in Stimuli and of the same ethnicity as subjects (Set FS-I),
- **Set 2**: familiar to the subjects in Stimuli and of the same ethnicity as subjects (Set FS-II) (redundant set of Set 1),
- **Set 3**: unfamiliar to the subjects in Stimuli and of the same ethnicity as subjects (Set US), and
- **Set 4**: unfamiliar to the subjects in Stimuli and of different ethnicity than subjects (Set UD).

Note that, one more combination, i.e. familiar to the subjects in Stimuli and of different ethnicity, is possible. However, due to the lack of participants satisfying this criteria, we have not been able to show study related to such a set.

### Stimuli, Design and Procedure

Each of the four sets consisted of 100 unique participants and the stimuli consisted of subjects of ID V1 dataset collected at IIIT-Delhi. Since the participants in Sets FS-I & FS-II and stimuli belonged to the same department in IIIT-Delhi, it ensured familiarity and same ethnicity factors. Set FS-I and Set FS-II were redundant in nature, as they were similar in terms of familiarity and ethnicity. However, having access to two groups with participants of same variable provided scope for more analysis in terms of the consistency of outcomes. To ensure the unfamiliarity factor in Set US, it consisted of participants from another city of a different state of India. As the two cities are far apart and no logical connection among subjects and participants was known, it was safely assumed that the participants in Set US were unfamiliar to the stimuli subjects. Since the participants in Set FS and Set US were from India, they were of the same ethnicity as the stimuli. Set UD consisted of participants of Chinese ethnicity, thus ensuring unfamiliarity and different ethnicity than that of stimuli. Table 2 summarizes the details regarding the number of participants and gender distribution in each set.

**Table 2:** Age and gender distribution of participants in the four sets.

Set	Overall		Male		Female		Gender Not Specified
	No.	Age 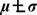	No.	Age 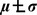	No.	Age 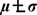	No.
Familiar, Same Ethnicity-1 (FS-I)	100	18.5 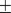 0.8	68	18.5 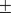 0.6	30	18.5 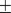 0.6	2
Familiar, Same Ethnicity-1 (FS-II)	100	20.5 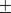 3.5	58	20.7 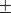 3.8	38	20.2 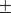 3.8	4
Unfamiliar, Same Ethnicity (US)	100	19.5 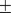 2.5	64	19.5 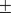 2.5	33	19.5 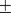 2.5	3
Unfamiliar, Different Ethnicity (UD)	100	23.6 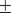 3.8	55	24.6 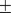 5.6	44	22.4 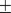 5.6	1

The results reported are mean values with standard deviation.

**Figure 4:**
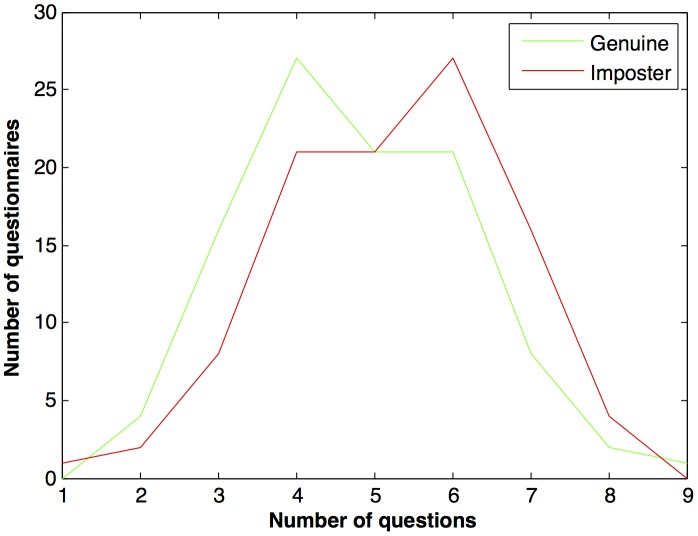
Distribution of genuine and impostor pairs in questionnaires.

Each participant was given a questionnaire containing eight face image pairs. The participants were supposed to mark them as “same person” or “not same person”. Optionally, the participants were also requested to write their age and gender. Each participant in a set was given a unique questionnaire. However, there were overlapping questions among different questionnaires. Therefore, 100 questionnaires were designed by randomly choosing genuine (same person) and impostor (different person) image pairs with equal priors. The pairs were drawn from a split that contained neutral and disguised face images pertaining to 40 subjects. The pairs for each questionnaire were selected with substitution, therefore an image pair could appear in multiple questionnaires; however it was made sure that no image pair was repeated in the same questionnaire. Thus, across 100 questionnaires, 436 unique image pairs were used. Figure 4 shows the distribution of genuine and impostor pairs in questionnaires. Note that the majority of questionnaires had an even mixture of genuine and impostor image pairs. Further, the face images were converted to gray scale and elliptical mask was applied to face images to make sure that no features other than facial cues could be used for recognition. All the face images were resized to 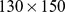 pixels which translated to 2.8 cm
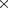
3.2 cm on a printed document of A4 size. One such example questionnaire is shown in Figure 2. The exact same set of 100 questionnaires was used for collecting responses from the participants of Set FS-I, Set FS-II, Set US, and Set UD.

One of the objectives of this research is to compare human evaluation with automated algorithms. Automated algorithms are generally evaluated in either face matching/verification or face identification scenarios. In face matching or verification scenario, an image pair is classified as match or non-match, whereas in face identification scenario a query image is compared with gallery/enrolled face images to predict the identity. For comparing the human and machine performance, it is essential that the comparison metric is same for both. Simulating identification scenario for human evaluation involves two challenges:

- First, the gallery images are to be shown to the subjects for *enrolling* them in their memory. However, this process becomes challenging with increasing number of gallery images.
- Identification performance of an automatic algorithm is measured in terms of cumulative match characteristics (CMC) curve, which requires to get ranked list of gallery images in sorted order of matching with the query image. Therefore, if human performance is to be compared with algorithms in identification scenario, the ranking is required to be generated by humans too. This is practically possible if number of gallery images is small. However, it is rather difficult, from experimental design and participants perspective, when the number of gallery images is large.

Further, existing research in human versus algorithm comparisons focuses on verification scenario [36], [37]; therefore this paper also focuses on the same. Apart from comparing, we also aim at incorporating the understandings from human cognition into an automated algorithm.

A mixed-subjects design was employed in which the *between-subjects* variables were familiarity (familiar or unfamiliar), ethnicity (same as stimuli or different from stimuli), and gender (male or female). The participants took part in only one of the four sets/Familiarity-Ethnicity combinations (Set FS-I, Set FS-II, Set US, and Set UD). The combination of Familiar-Different Ethnicity could not be evaluated as it is challenging to find such participants. The *within-subjects* variable was the amount of disguise on stimuli face images. The participants in each of the sets followed the same procedure, i.e. they were given a questionnaire containing eight face image pairs and they marked each pair as “same person” or “not same person”.

The evaluations are performed in terms of the False Accept Rate (
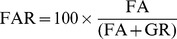
), Genuine Accept Rate (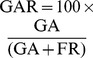), and Accuracy (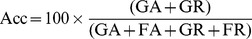), where 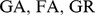 and 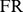 represent the number of genuinely accepted, falsely accepted, genuinely rejected, and falsely rejected pairs respectively. False accept means that a non-match pair is classified as a match pair and genuine accept means that a match pair is correctly classified. A face recognition is expected to achieve high GAR at low FAR.

The results of F-test with 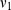 and 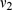 degrees of freedom are denoted as 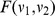, similarly, the t-test with 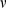 degrees of freedom is denoted as 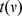. All the test results are reported with the corresponding 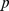-value. 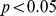, 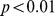, and 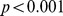 indicate moderately, strongly, and very strongly significant evidences respectively.

### Observations from Human Evaluation

The responses collected from participants of all the sets (Set FS, Set US, and Set UD) are used to compute the false accept rate, genuine accept rate, and accuracy. The major reason for evaluating the FAR and GAR along with accuracy is that accuracy does not provide information about GAR and FAR individually. Therefore, evaluating the performance in terms of GAR and FAR separately may help in understanding the efficiency of matching genuine and impostor pairs individually. The mean and standard deviations are reported in Table 3.

**Table 3:** Summary of human performance.

Set	FAR %( 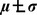 )	GAR %( 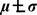 )	Accuracy %( 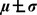 )
Set FS-I	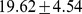	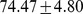	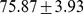
Set FS-II	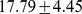	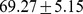	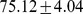
Set US	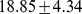	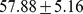	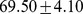
Set UD	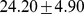	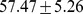	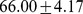

It is reported in terms of mean FAR, GAR and accuracy in each of the four sets.

Statistical tests are performed to further analyze these results. Three One-Way ANOVAs (Analysis of variance) are conducted to evaluate the statistical significance of FAR, GAR, and Accuracy. The results of these tests are as follows.

1. FAR (F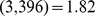, 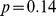),
2. GAR (F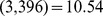, 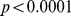), and
3. Accuracy (F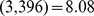, 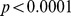).

This analysis of 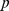-values shows that there is a significant difference in terms of GAR and accuracy with the corresponding 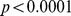 for both the statistics. However, there is no significant difference for FAR, since 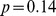. Post-hoc analysis is carried out using paired t-test to understand the 1) effect of familiarity, 2) effect of ethnicity, 3) effect of gender, 4) consistency between Set FS-I and Set FS-II, and 5) effect of specific disguise. The details of this analysis are provided below. The results and inferences of the statistical tests to understand the effect of familiarity, ethnicity, gender and consistency are summarized in Table 4.

**Table 4:** 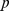
-values of statistical tests to understand the effect of each factor.

Factor	Sets Compared	FAR	GAR	ACC	Inference
Familiarity	FS-I & US	0.7829 ( 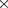 )	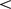 0.0001 ( 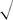 )	0.0035 ( 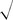 )	Unfamiliarity degrades GAR but not FAR
	FS-II & US	0.6856 ( 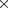 )	0.0022 ( 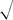 )	0.0061 ( 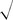 )	
Ethnicity	US & UD	0.0715 ( 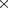 )	0.9103 ( 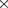 )	0.0789 ( 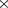 )	No additional degradation
Consistency	FS-I & FS-II	0.6878 ( 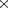 )	0.1025 ( 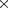 )	0.7199 ( 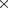 )	Both sets are consistent
Gender	FS-I (M) & FS-I (F)	0.1573 ( 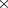 )	0.2420 ( 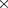 )	0.0171 ( 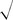 )	Female are better in Sets FS-I and FS-II. For other sets no significant difference is observed
	FS-II (M) & FS-II (F)	0.4529 ( 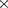 )	0.6801 ( 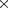 )	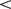 0.0001 ( 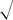 )	
	US (M) & US (F)	0.3776 ( 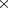 )	0.3785 ( 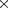 )	0.9535 ( 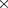 )	
	UD (M) & UD (F)	0.3535 ( 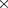 )	0.2737 ( 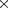 )	0.1524 ( 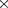 )	

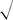
 represents that the corresponding statistical test show significant difference between the compared sets and 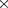 represents insignificant difference.

#### Effect of Familiarity

To evaluate the effect of familiarity for each of the three statistics i.e. FAR, GAR, and Accuracy, two paired t-tests are performed: 1) between Set FS-I and Set US and 2) between Set FS-II and Set US. In both cases, significant accuracy improvement is observed when the participants are familiar to the stimuli. The p-values for accuracy are reported as follows.

- Set FS-I and Set US: t(99) = 2.99, 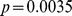

- Set FS-II and Set US: t(99) = 2.80, 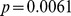

However, no significant difference is observed for FAR.

- Set FS-I and Set US: t(99) = 0.288, 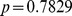

- Set FS-II and Set US: t(99) = -0.4060, 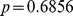

Further, GAR is observed to be different for both the cases

- Set FS-I and Set US: t(99) = 4.86, 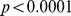

- Set FS-II and Set US: t(99) = 3.14, 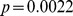.

It is observed that the best performance is achieved when the participants are familiar with the stimuli face and are of the same ethnicity. Interestingly, Sets FS-I & FS-II have the same FAR as Set US, but Set US has significantly lower GAR. This means that when participants are unfamiliar to stimuli, they tend to reject more genuine matches. From the observation regarding similar FAR in Set FS-I, FS-II, and US, one can claim that: if a pair has images of different individuals, an unfamiliar participant will classify it as “same person” with equal likelihood as a familiar participant. Moreover, the finding that “familiar faces are easier to match even if they are disguised” is equivalent to the similar finding for non-disguised faces [31]. Although, Douma et al. [25] did not find the effect of familiarity significant in recognizing disguised faces, note that our experimental procedure is different from their's. In [25], the participants were to *identify* the stimuli faces, whereas in this study the participants were to classify the stimuli image pairs as “same person” or “different persons”. The former involves the face identification scenario, where the performance is primarily a function of memory and internal representation of faces which is enhanced if the person is familiar. However, that is not the case with our study which involves face verification scenario as it enables us to compare human performance with algorithm. To summarize, *familiarity is an advantageous factor and unfamiliarity significantly degrades genuine accepts but not the false accepts.*

#### Effect of Ethnicity

To understand the effect of ethnicity, paired t-tests are performed between Set US (unfamiliar, same ethnicity) and Set UD (unfamiliar, different ethnicity). The participants in both these sets are unfamiliar to the stimuli subjects; Set US has the participants which are of same ethnicity as stimuli, whereas Set UD participants are of different ethnicity than stimuli. Among the unfamiliar participants, the one with different ethnicity does not result in significantly different accuracy (t(99) = −1.7757, 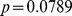). From further analysis in terms of FAR and GAR it is found that neither FAR (t(99) = 1.82, 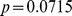) nor GAR (t(99) = −0.1129, 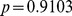) is significantly differing. This suggests that in the presence of disguise, different-ethnicity factors do not add to the reduction in performance due to unfamiliarity factor. Therefore, the other-race effect [32] does not significantly further deteriorate the performance of recognizing disguised faces if the participants are unfamiliar to stimulus. However, if the participant is of the same ethnicity as the stimulus, familiarity is an added advantage.

#### Effect of Gender

No specific effect of gender is observed, except on the accuracy of Set FS-I (t(96) = −2.427, 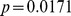) and Set FS-II (t(94) = −15.56, 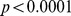) where female participants exhibit significantly better performance than male participants. However, even for these two sets no significant difference in FAR or GAR is observed. Similar observation regarding female superiority for face recognition has been studied in literature [43]. However, for disguised face recognition, this effect is observed only when the participants are familiar to stimuli faces and it disappears with absence of familiarity and/or difference in ethnicity.

#### Consistency between Set FS-I and Set FS-II

As we have access to two sets with the same familiarly and same ethnicity settings, it enables us to perform a consistency check, i.e. to evaluate similarity between the results of two sets with same design variables. We performed paired t-tests between Set FS-I & Set FS-II to analyze if there is any performance difference. Without much surprise, there is no significant difference in FAR (t(99) = 0.6878, 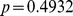), GAR (t(99) = 1.6481, 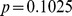), and accuracy (t(99) = 0.3596, 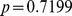). For comparison, the response of both the sets are illustrated in the form of a confusion matrix in Table 5. Thus, similar performance is observed in both the sets.

**Table 5:** Confusion matrix for comparing the consistency of Set FS-I and Set FS-II.

Confusion		Set FS-I	
Matrix		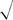	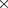
Set FS-II	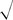	227	108
	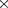	130	335

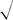
 and 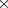 represent the genuine and impostor classified samples respectively. The numbers in every cell represent the co-occurrence of decisions (correct/incorrect). For example, 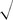 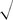 block shows that for 227 image pairs, participants in both Set FS-I and Set FS-II responded that they were genuine pairs.

#### Effect of Specific Disguises

**Figure 5:**
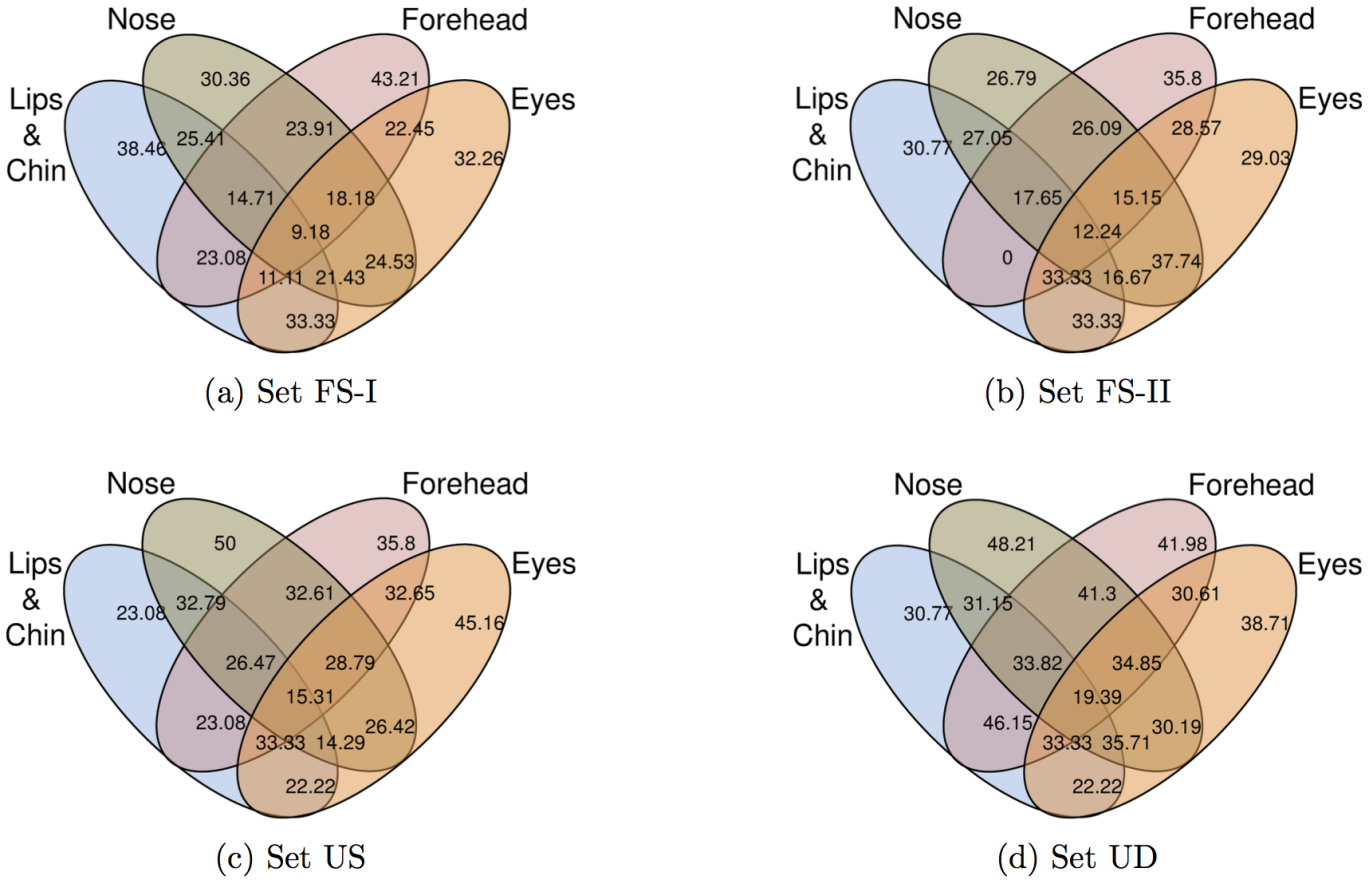
Effect of disguising individual facials parts and their combinations. The numbers represent the percentage of the misclassified face image pairs belonging to the corresponding disguise combination. For example, there are 31 image pairs with disguise on eye strips only, out of which 10 are misclassified by the participants in Set FS-I (a). This leads to the aforementioned incorrect classification fraction of 
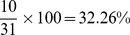
.

In this analysis, we focus on enhancing the understanding regarding the effect of specific kinds of disguises on face recognition performance. Human performance decreases when faces are disguised [23]. However, the effect of various kinds of disguises and their combinations is not yet well explored. The presence of disguise on certain facial parts can corrupt or occlude the partial face information thus degrading the face recognition performance. We divide the face image into uniform 5
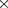
5 grids and label the first, second and third rows as forehead, eyes, and nose regions respectively. The remaining two rows taken together are labeled as lips and chin region. From manual annotation of every rectangular patch of the grid, we have information regarding which patch contains disguise. The disguised patches are referred to as non-biometric patches. A region is considered to contain disguise if more than half of the patches in that region are non-biometric patches. Since the face images are divided into four non-overlapping regions, there can be (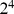 = )16 combinations of disguised regions. These combinations can be represented in the form of a 4 set venn diagram. Figure 5 represents such a venn diagram representing the percentage of incorrectly classified face image pairs belonging to each disguise combination. Figure 5(a), (b), (c), and (d) represent venn diagrams pertaining to Set FS-I, Set FS-II, Set US, and Set UD respectively. Note that in the ideal case, all the numbers in the venn diagram would be zero, i.e. none of the face image pairs belonging to any of the disguise combination is incorrectly classified. The key observations are as follows.

- Intuitively, the accuracy of disguised face recognition should reduce with increase in the amount of disguise. However, consistently for all four sets, considerably high errors are reported even when only a single kind of disguise is present (see the *only nose, only eyes, only forehead*, and *only lips* in Figure 5). This may be due to the fact that when an image-pair contains only one kind of disguise, one or both the face images contain similar kind of disguise. Also from the database section it can be noted that the number of disguise accessories applicable to each facial part, such as eye-glasses and bandanas, are limited in number. Therefore, variations in accessories disguising each facial part are limited. As the disguise accessories are encoded as part of the overall presentation in human perception [23], use of 1) same kind of disguise accessories among different users and 2) different kinds of disguise accessories on the same user might be leading to higher error rates.
- In the other regions of the venn diagram i.e. with multiple disguises, images in the face image pairs can have disguise accessories affecting different facial feature(s), therefore the argument regarding the similar disguise accessories cannot be applied to them.
- Intersecting areas of venn diagrams corresponding to facial hairs and wigs i.e. forehead-nose and forehead-nose-lips-and-chin also yield considerably high error rates, implying that the co-occurrence of wig and mustache (and beard) makes it challenging to match two faces. Though, the negative impact of combination of disguises is less prominent than that of disguise in only one part, there is a steady trend of its increased impact with increase of challenging factors, i.e. Set FS 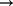 Set US 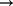 Set UD.

### Anāvṛta: Proposed Face Recognition Approach

From the human evaluation study presented above, it is clear that use of disguise accessories degrades the recognition performance. This is majorly because disguise accessories get encoded as a part in the overall presentation [23]. Moreover, use of disguise accessories can also reduce the uniqueness of subjects. From automated face recognition point of view, Pavlidis and Symosek [44] have suggested that detecting disguise is necessary to efficiently recognize disguised faces. Therefore, using learnings from the human analysis, we develop the following hypothesis for automated face recognition:

“The facial part or patches which are under the effect of disguise (or occluded in most of the cases), are the least useful for face recognition, and may also provide misleading details. It is this misrepresentation that a person uses to hide his/her own identity and/or to impersonate someone else.”

**Figure 6:**
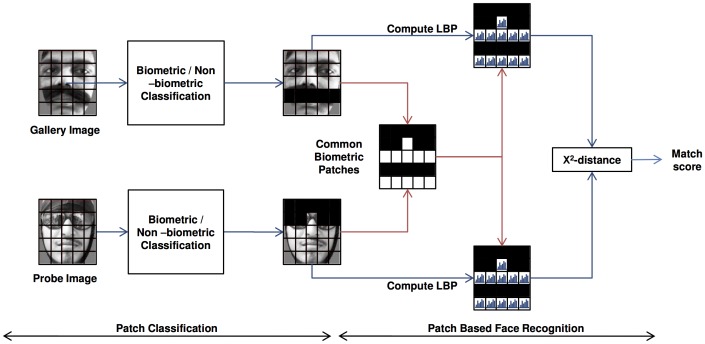
Illustrating the steps involved in the proposed face recognition framework.

Building upon this intuition, we propose a framework, termed as Anāv
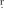
ta, for recognizing faces with variations in disguise. As illustrated in Figure 6 there are two stages in the proposed framework:

1. **Patch Classification**. It comprises dividing face image into patches and classifying them into *biometric* or *non-biometric* classes.
2. **Patch based Face Recognition**. Biometric patches are matched using local binary pattern (LBP) based face recognition algorithm.

#### Patch Classification

In human cognition research, Gosselin and Schyns [45] have proposed a technique to identify relevant facial regions for recognition which shows that certain facial parts are more important than others for recognition. In automated algorithm literature, several researchers have proposed patch or part-based face recognition [6], [46]–[48] and evaluated the performance of individual parts for face recognition. De Marsicso et al. [47], [48] proposed a solution based on local information where each facial part is used separately as input; the scores obtained by matching each part are fused to obtain final scores. Moreover, the mechanism for self-tuning the subsystems for matching individual parts was also proposed. To the best of our knowledge, [49], [50] are the only works in literature which use occlusion detection to enhance the recognition performance. In applications such as law-enforcement, analyzing the patches to determine whether they are genuine facial regions or accessories is very important. The proposed patch classification algorithm therefore aims to classify the patches into biometric and non-biometric classes.

- **Biometric patches** are those facial parts that are not disguised; and hence they are useful for recognition.
- **Non-biometric patches/artifacts** are facial parts that are disguised. These patches may reduce the performance and should be avoided as far as possible.

The patch classification algorithm has two steps: feature extraction and classification.

1. **ITE Feature Extraction.** It is our assertion that some of the non-biometric patches or occlusions, such as hair and artificial nose, can be distinguished using texture information, while some others, such as scarves and sunglasses, can be distinguished using their intensity values. Therefore, the proposed algorithm uses a concatenation of texture and intensity descriptors as input feature. As shown in Figure 6, the algorithm starts with tessellating the face image. Input face image 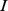 is first divided into non-overlapping rectangular patches 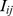, 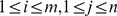, where 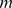 and 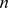 are the number of horizontal and vertical patches respectively. The intensity and texture descriptors are computed for all the patches using the intensity histogram and Local Binary Patterns (LBP) algorithm [6] respectively. The proposed descriptor is termed as the *Intensity and Texture Encoder* (ITE). For a patch 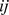 of an image 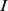, ITE is defined as
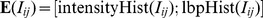
(1)where 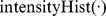 represents the histogram of an intensity image and 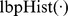 represents the LBP histogram. We use basic LBP operator with 8 sampling points, that produces 256 dimensional feature vector for each patch. Intensity histogram consists of 256 bins, resulting in a feature vector of the same dimension.
2. **ITE Feature Classification.** The ITE features can, potentially, be classified using any of the generative or discriminative classification techniques. Our observation of biometric and non-biometric patches shows that the set of biometric patches is well defined and can be modeled efficiently. However, due to the variety of accessories that can be used for disguise, non-biometric patches have an exhaustive population set which is difficult to model using a limited training database. Therefore, in this research, we have used Support Vector Machine (SVM) [51], a discriminative classifier, for classifying biometric and non-biometric patches.
  An SVM model is learned from the ITE descriptors of all the patches from training images (which are annotated manually). This model is used to classify the patches from the testing data. For every patch, a score 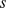 is computed using SVM. A patch is classified as biometric, if the score is less than the threshold 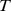, i.e. 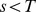; and if score is equal to or greater than the threshold, i.e. 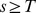, the patch is classified as non-biometric. Accordingly, a flag variable 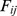 is generated, which represents whether the patch is classified as biometric or non-biometric. The flag value of every patch is then combined to generate the flag matrix, 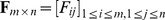, using Eq. 2.
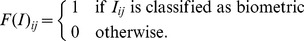
(2)
  ITE features of images patches are classified using trained SVM model.

#### Patch based Face Recognition

Let 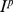 be the probe image which is to be matched with the gallery image 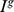. The corresponding flag matrices 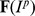 and 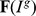 are generated using Eq. 2. Here, it is possible that for some gallery patch, 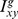, which is classified as biometric, the corresponding probe patch, 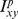, is classified as non-biometric. In other words, 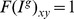 and 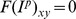, or 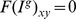 and 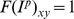. This renders the particular patch of gallery image not useful for recognition because the corresponding patch from the probe image is under disguise effect and matching a biometric patch with a non-biometric patch may lead to incorrect information.
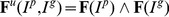
(3)


The patch classification algorithm explained in previous Section classifies the patches into biometric and non-biometric, and Eq. 3 provides information that *for a given gallery-probe pair, which patches should be used for face recognition*. Note that, in order to take advantage of patch classification, the face recognition approach has to be patch-based. Therefore, we propose to use LBP [6] which is one of the widely used patch-based descriptors for face recognition. If 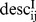 represents the LBP descriptor of 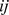 patch of image 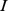, and the 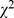-distance between two LBP descriptors is represented as 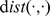, then the distance 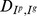 between two images, 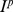 and 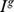, is calculated as:
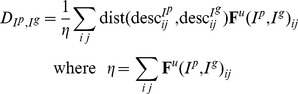
(4)and 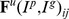 is obtained using Eq. 3.

### Results of the Proposed Algorithm

This section demonstrates the results of the proposed face recognition framework which includes the patch classification algorithm and LBP based face recognition, along with its comparison to SRC andCOTS. We also compare the results of proposed algorithm with the results of human evaluation results.

**Figure 7:**
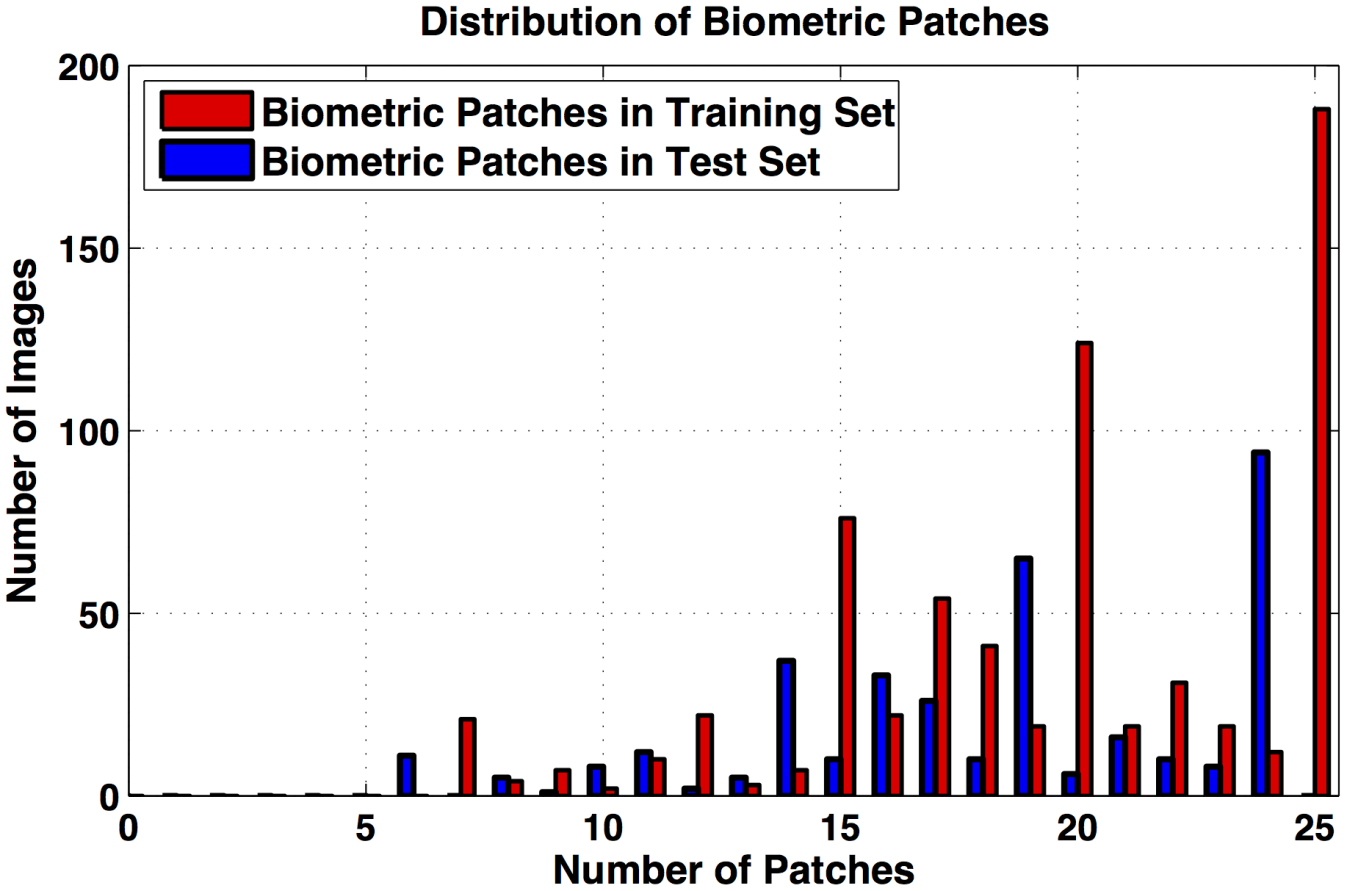
The distribution of biometric patches in the training and test sets.

All the images in the database are divided into 5
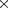
5 non-overlapping rectangular patches of size 26
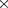
30 pixels. Every patch is manually annotated as biometric or non-biometric to create the ground truth for training as well as evaluation. If more than half of the patch is covered with accessories, it is annotated as a non-biometric patch. Images of randomly chosen 35 subjects form the training set and the images from the remaining 40 subjects are used for testing. The training set thus contains 8050 patches (322 images
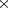
25 patches), out of which 6324 patches are biometric and 1726 patches are non-biometric. Similarly, the testing set comprises 8975 patches (359 images
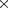
25 patches) amongst which 6944 are biometric and 2031 are non-biometric. Depending on the disguise accessories used, the number of biometric patches in every image vary. Figure 7 shows the distribution of (annotated) biometric patches in the training and testing splits.

#### Patch Classification using ITE

**Figure 8:**
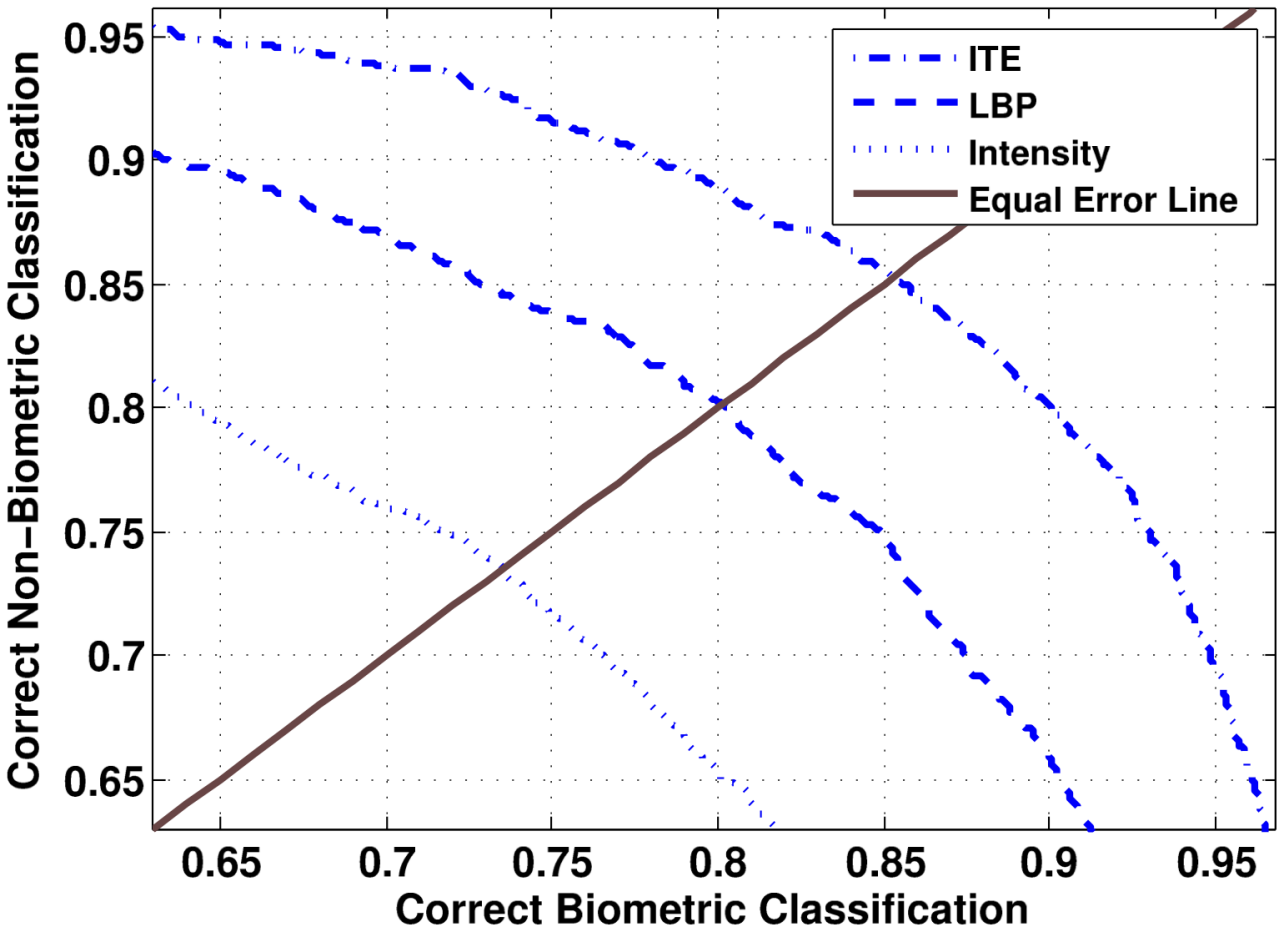
ROC curves for patch classification.

As explained earlier, for each patch, the ITE features are computed using Eq. 1; and min-max normalization is performed to map the values in the interval 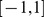. The normalized descriptor is provided as input to SVM with Radial Basis Function kernel for patch classification. The kernel parameter and misclassification cost are estimated using grid search along with 5-fold cross validation. In grid search, parameters that provide the maximum training accuracy are considered as optimum. Since ITE is a concatenation of LBP and intensity values, the efficacy of ITE is compared with LBP and pixel intensity values. LBP histograms, intensity histograms, and ITE histograms are computed and provided as input to SVM separately for classification. Receiver Operating Characteristics (ROC) curves for patch classification representing the results of these experiments are shown in Figure 8. Note that, ITE provides better results compared to either texture or intensity information for patch classification. This supports our hypothesis that *concatenation of texture and intensity features should yield better patch classification results*.

#### Performance Evaluation of Anāvṛta

The output of patch classification yields biometric patches which are used for feature extraction and matching. For evaluating the proposed face matching approach, the testing set is divided into two parts: gallery and probe. For each subject, one neutral face image, and four other randomly selected images are taken as gallery and the remaining images constitute the probe/query set. Hence, there are total 200 gallery images and 159 probe images. We have performed experiments with five random cross validation trials. The experiments are performed in verification mode and the results are reported in terms of ROC curve and verification accuracy at 0.1%, 1.0% and 10% False Accept Rate (FAR). To understand the importance and effectiveness of performing patch classification, we performed the following three experiments.

1. Face recognition with biometric patches is classified using ITE and SVM classifier,
2. Face recognition with manually annotated biometric patches, and
3. Face recognition with all the patches (normal LBP approach)

**Figure 9:**
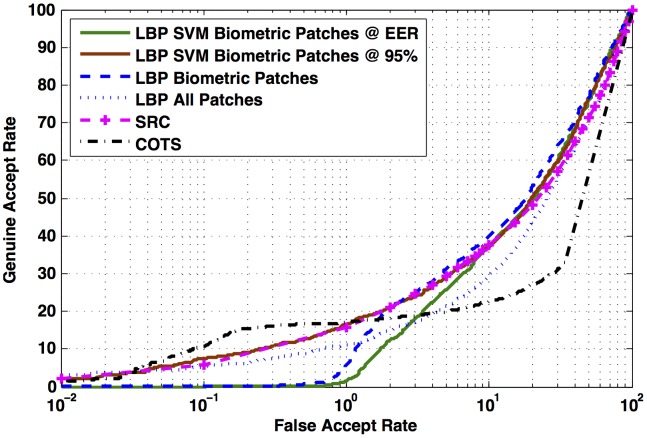
The results of the proposed face recognition framework using LBP descriptor.

The results of face recognition are shown in Figure 9. For FAR
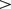
1%, using only ground truth biometric patches results in better accuracy than using all the patches for face recognition. The performance of the proposed framework depends significantly on the performance of the patch classification algorithm. Intuitively, rejecting a non-biometric patch is less benefitting than the loss incurred by wrongly rejecting a biometric patch. From the ROC curve of patch classification shown in Figure 8, it can be analyzed that at equal error rate (EER), 15% of the biometric patches are being misclassified. show that the performance of face recognition reduces when the threshold of patch classification is chosen at EER. The ROC curves in Figure 9 show that the performance of face recognition reduces when the threshold of patch classification is chosen at EER. This may be attributed to the reduction in the number of biometric patches used for face recognition at that threshold. However, for 95% correct biometric patch classification (Figure 8), even though the number of correctly classified non-biometric patches decreases, the face recognition algorithm is receiving more biometric patches as input and the proposed face recognition framework yields better performance than simple LBP based approach. This supports our hypothesis that not using non-biometric patches for recognition can result in better accuracy.

#### Comparison with COTS and Sparse Representation

In this section, we present a comparison with FaceVacs commercial off-the-shelf face recognition system (referred as COTS) and sparse representation classifier (SRC) [8]. Note that, SRC is a recent technique in literature for addressing occlusion/disguise. In SRC, the residual is considered as the dissimilarity measure of the gallery-probe pair. For evaluating the performance of the proposed framework, we have utilized all the gallery and probe images irrespective of the information content or image quality. However, COTS used in this research has inbuilt algorithms for quality assessment and enrollment. The thresholds for enrolling a gallery image are very strict whereas for probe images, it is relaxed. Out of the 200 gallery images, COTS enrolled approximately 60% of the gallery images and the remaining images were considered as *failure to enroll* whereas all the probe images were processed successfully. It is also observed that if the face image does not contain any non-biometric patch, then the probability of getting enrolled in the COTS is higher. However, for a fair comparison, we have overridden the COTS to include all 200 images in the gallery. Figure 9 and Table 6 demonstrate the results of COTS and SRC along with the proposed algorithm.

**Table 6:** Results from automated algorithms.

Approach	Verification Accuracy @ FAR		
	0.1%	1.0%	10%
SRC	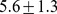	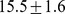	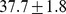
COTS	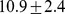	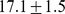	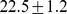
Proposed	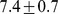	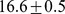	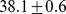

Genuine accept rates and their standard deviations at different false accept rates of the proposed approach along with comparison to COTS and SRC.

For face databases captured in constrained environment with cooperative users, face recognition algorithms yield high GAR, and it increases with increase in FAR [21]. However, this kind of trend is not found on this dataset with any of the three algorithms, thereby, showing the challenging nature of the database itself. It can be observed that COTS is not able to classify the faces under disguises very well as corresponding GAR does not increase much with increase in FAR. For lower FAR (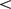
0.05%), all the approaches shown in comparison exhibit very poor performance. From approximately 0.2% till 5% FAR, the verification rate of COTS improves from 16% to 20% GAR. This may be attributed to COTS discarding many samples due to internal minimum quality criterion. For the same range of 0.2% to 5% FAR, the proposed approach yields up to 30% GAR. For almost whole range of FAR, the proposed approach is comparable to SRC. As shown in Table 6, although the performance reported by the proposed approach is not as high as it is usually reported in face recognition literature, it outperforms one of the state-of-art commercial systems and is comparable with a widely used technique (i.e. sparse representation).

In the evaluation of the proposed algorithm, it is observed that the performance of local (patch-based) face recognition algorithm can be improved by rejecting the face patches that contain disguise. Strict rejection of non-biometric patches leads to lower GAR at lower FAR. However, as discussed earlier a flexible patch classification at 95% correct biometric patch classification exhibits higher GAR even at lower FAR. Moreover, for FAR
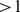
% the proposed automated algorithm outperforms the COTS which ends up rejecting large number of disguised face images which do not match its minimum criteria for processing. Although, the proposed algorithm equates to SRC [8] and outperforms COTS, the overall performance of 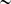
17% GAR at 1% FAR compared 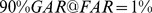 with very high accuracy that is usually reported for face verification of frontal non-disguised faces [21], suggest that significant amount of research is required to efficiently mitigate the effect of disguise variations.

### Comparison of Human Responses with Automated Algorithms

**Figure 10:**
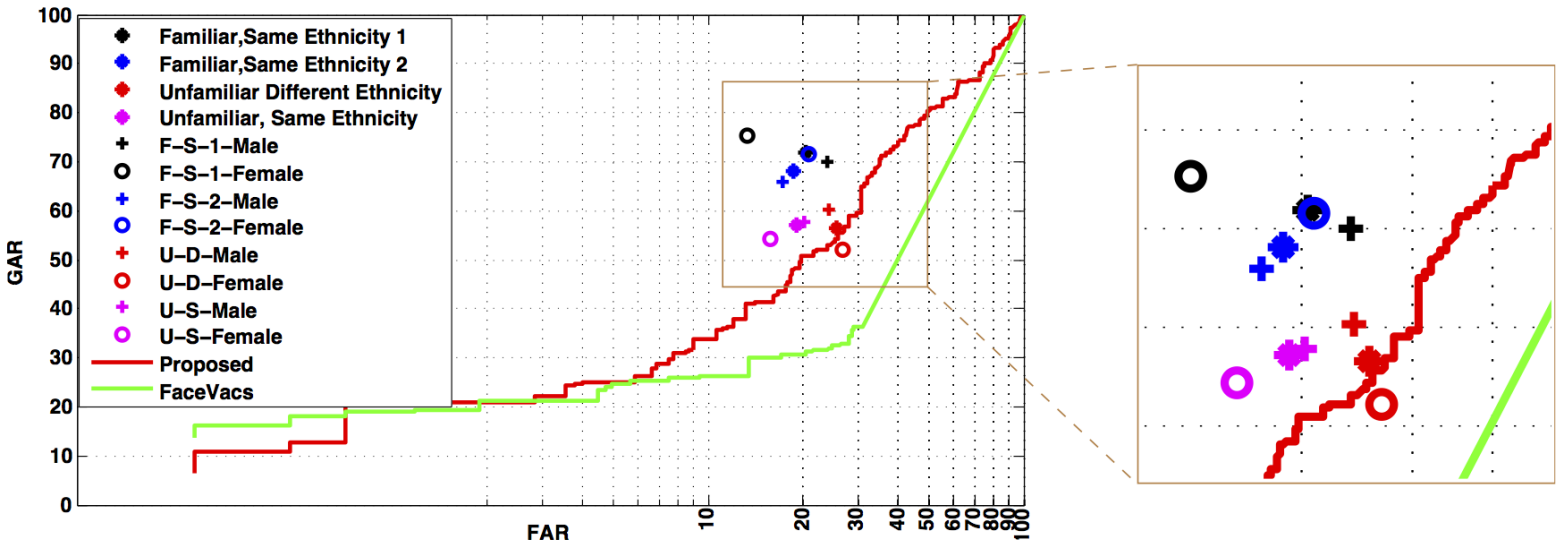
Performance of disguised face recognition by humans, with respect to familiarity and ethnicity. Analyzing the effect of familiarity and ethnicity on the performance of disguised face recognition by humans.

As opposed to automated algorithm where for every image pair a match score is computed and compared with decision threshold to estimate the accuracy, human evaluation directly records their final decision. Therefore, for the automated algorithm ROC can be drawn by varying the threshold, whereas only a point (FAR-GAR pair) can be obtained on ROC from the human evaluation. Figure 10 represents the performance of all four Sets along with respective ROCs of the proposed automated algorithm and COTS. The key observations are as follows.

- The performance of Set FS (familiar, same ethnicity) is better than the one reported with automated algorithms (proposed and COTS).
- The ROC curve of the proposed algorithm passes through the performance point pertaining to Set UD. This is probably due to the fact that the automated algorithm does not encode familiarity or ethnicity, leading to no performance bias because of these two factors. Thus, proposed automated algorithm is comparable to humans recognizing unfamiliar subjects of different ethnicity. O′Toole et al. [37] have also observed that difference between the performance of humans and state-of-the-art face recognition algorithms were analogous to differences between humans recognizing familiar versus unfamiliar subjects. Researchers have also suggested that mental representation of familiar faces [52] helps make the familiar face recognition efficient compared to unfamiliar face recognition. If the machine counterpart of the mental representation is not incorporated somehow, the algorithms would face challenges similar to that of unfamiliar face recognition by humans.
- Although, FAR from human evaluations are smaller than that from automated algorithm, human performances exhibit considerably higher FARs ranging from 10%–30%.
- The proposed approach is a local approach and does not encode the holistic facial features whereas humans have access to both local and holistic facial information. Note that, we ended up using the local approach as the holistic features can be corrupted by local disguises. The proposed local approach (ITE based patch classification+LBP based recognition) does improve performance over traditional local approach (LBP based recognition). However, the improved performance is only equivalent to the worst of human performance (Set UD) which favorably underlines the likely use of holistic facial features by humans. Therefore, simultaneous use of holistic and local facial features can lead to superior disguised face recognition performance.
- Our study on human evaluation suggests that ethnicity and familiarity of faces can greatly affect the face recognition performance. incorporating this information in face recognition algorithms can also provide improved matching accuracy.

## Conclusion and Future Work

This paper presents a study on the effect of ethnicity and familiarity on the performance of face recognition in presence of disguise variations. The recognition accuracy of familiar-and-same-ethnicity subjects is found to be significantly better than that of unfamiliar-and-different-ethnicity. It is observed that if the ethnicity is same; unfamiliarity does not significantly affect correct rejection. Our experiments do not show any evidence of decrease in cross-ethnicity face recognition under disguise. We also observe that use of similar disguise accessories account for considerably high error rates.

Encoding the understanding from human evaluation, we propose an automated face recognition algorithm. The proposed algorithm consists of the ITE based patch classification (in biometric/non-biometric classes) and LBP based face recognition applied on classified biometric patches. The performance is evaluated on the IIIT-Delhi disguise database pertaining to 75 subjects. The proposed algorithm outperforms a COTS and classical LBP based face recognition. The performance of the proposed algorithm is comparable with SRC and the human performance of unfamiliar-and-different-ethnicity. Though we report performance improvement with the proposed algorithm, it is still an open research problem. The results of automatic algorithms are similar to unfamiliar face recognition performance of humans and therefore there is a scope for extending this research in the direction of both cognitive as well as automatic face recognition. As a future research direction, we plan to encode and incorporate the concept of familiarity in automatic algorithms which may improve the performance. Further, we also believe that the study of how disguising individual facial parts affect representations of faces might lead to better solutions to mitigate these variations.

## Supporting Information

Supporting Information S1 (PDF)
