# Supplementary material for: Recognizing Disguised Faces: Human and Machine Evaluation
Source: PLoS One. 2014 Jul 16;9(7):e99212. doi: 10.1371/journal.pone.0099212 (PMC4100743; doi:10.1371/journal.pone.0099212)
Supplement: Supporting Information S1 — (PDF) [file pone.0099212.s001.pdf]

**Image**	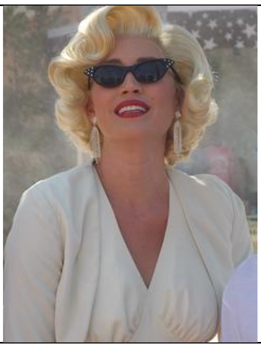	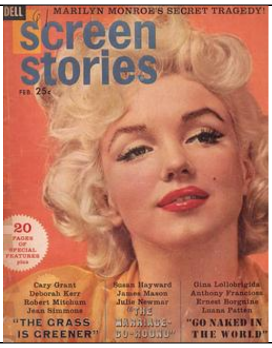	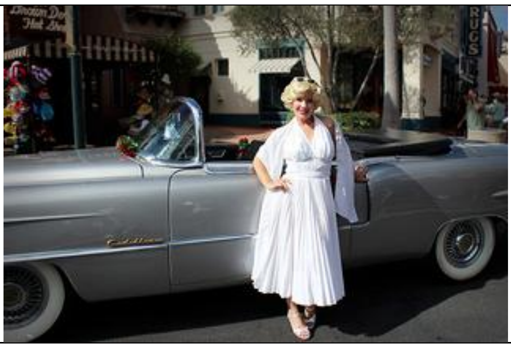
**URL**	http://www.flickr.com/photos/11325321@N08/7846433220/sizes/o/in/photostream/	http://www.flickr.com/photos/67861164@N05/7246988794/sizes/o/in/photostream/	http://www.flickr.com/photos/greyloch/8585849790/sizes/o/in/photostream
**License**	CC BY 2.0	CC BY 2.0	CC BY-SA 2.0
**Username and URL of creator**	starrynight_012<br><br>http://www.flickr.com/photos/11325321@N08/	RockyandNelson<br><br>http://www.flickr.com/photos/67861164@N05/	greyloch<br><br>http://www.flickr.com/photos/greyloch/
